# Prevalence of increased risk of type 2 diabetes in general practice: a cross-sectional study in Norway

**DOI:** 10.1186/s12875-023-02100-x

**Published:** 2023-07-20

**Authors:** Hilde Kristin Refvik Riise, Marit Graue, Jannicke Igland, Kåre I. Birkeland, Beate-Christin Hope Kolltveit

**Affiliations:** 1 Department of Health and Caring Sciences, Western Norway University of Applied Sciences, P.O. Box 7030, N-5020 Bergen, Norway; 2 Department of Global Public Health and Primary Care, University of Bergen, Bergen, Norway; 3 Department of Transplantation Medicine, Oslo University Hospital, Oslo, Norway; 4 Institute of Clinical Medicine, Faculty of Medicine, University of Oslo, Oslo, Norway; 5 Vossevangen Medical Center, Voss, Norway

**Keywords:** FINDRISC, General practice, Type 2 diabetes, Risk assessment, Prevalence, Prevention

## Abstract

**Background:**

Type 2 diabetes (T2D) is a global public health problem, but the onset can be delayed or prevented with adequate intervention in individuals with increased risk. Therefore, a major challenge in general practice is to identify individuals at risk of diabetes. However, limited knowledge is available about the prevalence of high diabetes risk individuals in a primary care population. In a cohort of consecutive patients in general practice we examined the prevalence of known diabetes and estimated risk of diabetes using The Finnish Diabetes Risk Score (FINDRISC) calculator, by sociodemographic and clinical characteristics.

**Methods:**

This study was a cross-sectional study conducted in four general practices in Western and Eastern Norway. A total of 1682 individuals, 20–80 years of age, were assessed for eligibility from May to December 2019. We excluded patients who actively declined participation (*n* = 112), were lost because of various organization challenges (*n* = 103) and patients who did not fulfil the inclusions criteria (*n* = 63). Diabetes prevalence and prevalence of individuals at risk of T2D with 95% confidence intervals (CI) were estimated for the total sample, by age group and for men and women separately. We tested for differences between groups using t-test for continuous variables and chi-square test (Pearson Chi-Square) for categorical variables.

**Results:**

Of 1404 individuals, 132 reported known diabetes, yielding a prevalence of 9.9% (95% CI 8.4–11.6). Among participants without a known diagnosis of diabetes, the following estimates of elevated risk assessment scores were found: FINDRISC score ≥ 11 32.8% (95% CI 30.3–35.4) and FINDRISC ≥ 15 10.0% (95% CI 8.6–11.9). Comparable results were found between the sexes.

**Conclusions:**

Detection of unknown diabetes and individuals with increased risk, is of high public health relevance for early implementation of preventive measures aimed to reduce the risk of diabetes and its complications through lifestyle modification. A simple, non-expensive questionnaire, such as FINDRISC, may be valuable as an initial screening method in general practice to identify those in need for preventive measures.

**Supplementary Information:**

The online version contains supplementary material available at 10.1186/s12875-023-02100-x.

## Introduction

General practice represents an important venue for addressing diabetes prevention, however, the knowledge on the prevalence of individuals at risk of type 2 diabetes (T2D) in general practice is limited. The prevalence of diabetes is steadily increasing worldwide, and the number of adults affected by diabetes is estimated to be around 462 million, corresponding to 6.3% of the world`s population [1]. Approximately 90% are due to T2D [1]. The prevalence of diabetes in Norway is estimated to 7.5% with known diabetes [2–4]. A recent study [5] of all inhabitants ≥ 20 years of age in the Trøndelag Region in Norway found a diabetes prevalence of 6.0%, of which, 11.1% were previously undiagnosed.

Several studies have shown that the onset of T2D can be markedly postponed by preventive lifestyle modifications [6, 7]. Increase in T2D prevalence worldwide may be attributable to a wide range of potential factors, the most important available for preventive measures being overweight, obesity and lack of exercise, that result in a spectrum of metabolic complications and cardiovascular disease [8–12].

The long-lasting asymptomatic diabetes stage represents a challenge as diabetes-related complications may develop during this time, and which further increases the risk of morbidity and mortality [13]. Prediction tools may therefore be important to identify individuals at high risk of developing T2D, as high-risk individuals may considerably lower their risk of diabetes through lifestyle interventions [14–16]. Prediction tools constitute an easy, non-invasive, and inexpensive approach to the assessment of an individual’s risk of T2D and may be used in general practice as well by the public e.g., through websites for healthy behaviors or at visits to the pharmacies to identify those in need for preventive measures. The Finnish Diabetes Risk Score (FINDRISC) is a well-known tool for risk assessment [17]. FINDRISC requires no laboratory testing and has been validated in multiple populations [18–22], including Norway [18]. However, in the Norwegian population, the validity of FINDRISC to predict the risk of diabetes among people with FINDRISC ≥ 15 has been questioned. The cut-off point for elevated risk is found to be substantially lower than originally assumed [18], suggesting that a lower FINDRISC cut-off point may be more appropriate.

Given the trend for a rapid increase in the number of people with diabetes worldwide, further studies are needed to determine the prevalence of people at risk of developing T2D in general practice. Also, complex and demanding issues are frequent in general practices, highlighting the need for studies examining the usability of tools for risk-assessment to promote better risk-factor management among people with risk of T2D. In the current study we aimed to; 1) examine the sociodemographic characteristics of patients in different risk categories of FINDRISC, 2) examine the clinical characteristics of patients in different risk categories of FINDRISC, 3) examine, by age and sex, the prevalence of patients in general practice with increased risk of developing T2D using the FINDRISC tool, and 4) examine, by age and sex, the prevalence of patients in general practice with T2D.

## Methods

### Study design and study population

The current study was a cross-sectional study conducted in four general practices in Western and Eastern Norway (Vestland and Viken counties). We invited all persons 20–80 years of age present in the waiting room area during the data collection period (from May to December 2019) at each of the four sites to attend the study. A total population of 1682 individuals were approached and assessed for eligibility. We consider that this cohort of consecutive patients is reasonably representative for people seeing their general practitioner (GP) for consultations, but not of the general population. The exclusion criteria were severe co-morbidity, major psychiatric disorder, severe cognitive deficiency and pregnancy. After excluding patients who actively declined participation (*n* = 112), were lost because of various logistic problems (*n* = 103) and patients who did not fulfil the inclusions criteria (*n* = 63), 1404 (83.5% participation rate) individuals participated.

### Data collection

In the current study, all participants filled out a questionnaire consisting of 1) FINDRISC to estimate the prevalence of individuals at risk of developing T2D (FINDRISC score ≥ 15 and/or body mass index (BMI) > 30), and prevalence of known diabetes (self-reported), 2) lifestyle and health data such as physical activity, smoking, BMI, and use of anti-hypertensive drugs, and 3) socio-demographic data such as sex, age, marital status, educational level and occupation. A study nurse was available at all sites to recruit individuals and to offer assistance with filling out the questionnaire. The study nurse did also assist with measuring weight, height and waist circumference.

FINDRISC, originally developed in a Finnish study population followed from 1987–1997, is a questionnaire that can be used as prediction tool to identify people at risk of developing T2D. It includes questions about age, BMI, physical activity, vegetable & fruit intake, medical treatment of hypertension, history of hyperglycemia and family history of diabetes. In the original version of FINDRISC, a risk score of 0–14 points implies a low to moderate risk of diabetes (1–17% chance of diabetes over 10 years) while 15–20 points imply a high risk of diabetes (33% risk of diabetes over 10 years. In a revised version of FINDRISC an additional level has been added and are defining a score of 20–26 as a very high risk of diabetes (50% risk of diabetes over 10 years) [17, 23].

### Exposure and outcome measures

Sociodemographic data were defined as follows; education (primary/middle school, secondary education, university/college ≤ 4 years, university/college > 4 years or other), marital status (married/cohabitant or other (divorced, single, widow/widower)), living situation (living with others or living alone), and work situation (working full-time, working part-time, retired or other). Lifestyle and health data were defined as follows; physical activity at least 30 min a day (yes/no), smoking (never smoked, smoke daily now or have smoked sometimes/daily or smoke sometimes now), BMI (< 25, 25–30 or > 30), have used anti-hypertensive drugs (yes/no), family history of diabetes (biological parents/siblings/children, grandparents/aunt/uncle/cousin or no). T2D was defined as self-reported diabetes. FINDRISC score was used as a continuous variable (0–26) or categorized in three different ways; < 11 versus ≥ 11, < 15 versus ≥ 15, and < 7, 7–10, 10–14, 15–19, ≥ 20. These categories are in line with former studies [17, 18], and enables comparison with previous findings. We use the term “at increased risk” throughout the text, indicating moderate to high risk of T2D.

### Statistical analyses

Descriptive statistics were presented as frequencies and percentages for categorical variables and means and standard deviations (SD) for continuous variables. To test for differences in sociodemographic and clinical measurements between different FINDRISC categories we used t-test for the continuous variables and a chi-square test (Pearson Chi-Square) for the categorical variables.

Diabetes prevalence and prevalence of individuals at risk of T2D with 95% confidence intervals (CI) were estimated for the total sample, by age group and for men and women separately. When calculating the prevalence of individuals at risk of T2D men and women with a known diabetes diagnosis were excluded.

The level of significance was defined as < 0.05 and STATA 16 was used in all analyses.

## Results

### Characteristics of the study population

Sociodemographic and clinical characteristics according to FINDRISC score are shown in Table 1. In brief, persons with FINDRISC score ≥ 15 had lower educational level compared to those with FINDRISC < 15, and a smaller percentage were married/cohabitant and working full-time. Compared to those with FINDRISC score < 15 men and women with FINDRISC score ≥ 15 reported less favourable health; they had higher BMI and waist circumference, and they also exercised less and ate less vegetables/fruit/berries. Comparable results were found when using a lower cut-off of FINDRISC score (11 instead of 15); the higher FINDRISC score, the less favourable health. Overall, 96.5% of the participants were Norwegians, leaving 3.5% with a different ethnic background (not reported in the table).

**Table 1:** Sociodemographic and clinical characteristics according to FINDRISC^†^ score in 1404 Norwegians in general practice

		**FINDRISC**^a^** score**					
	**T2D (*n* = 132)**	** < 11 (*n* = 855)**	** ≥ 11 (*n* = 417)**	***P*-value**^†^	** < 15 (*n* = 1143)**	** ≥ 15 (*n* = 129)**	***P*-value**^†^
**Age, mean (SD)**	62.5 (11.6)	48.8 (16.4)	61.1 (12.6)	< 0.001	51.6 (16.4)	63.4 (11.3)	< 0.001
**Gender**				0.736			0.612
Male, n (%)	65 (49.2)	393 (45.9)	187 (44.8)		523 (45.8)	56 (43.4)	
Female, n (%)	67 (50.8)	463 (54.2)	230 (55.2)		620 (54.2)	73 (56.6)	
**Marital status**^a^				0.984			0.503
Married/cohabitant, n (%)	95 (72.0)	637 (75.3)	310 (75.2)		853 (75.6)	94 (72.9)	
Other, n (%)	37 (28.0)	209 (24.7)	102 (24.8)		276 (24.5)	35 (27.1)	
**Educational level**				0.001			0.552
Primary/middle school, n (%)	37 (28.0)	135 (15.8)	97 (23.3)		205 (17.9)	27 (20.9)	
Secondary education, n (%)	63 (47.7)	386 (45.2)	201 (48.2)		523 (45.8)	64 (49.6)	
University/college ≤ 4 years, n (%)	20 (15.2)	224 (26.2)	83 (19.9)		279 (24.4)	28 (21.7)	
University/college > 4 years, n (%)	11 (8.3)	89 (10.4)	29 (7.0)		110 (9.6)	8 (6.2)	
Other, n (%)	1 (0.8)	21 (2.5)	7 (1.7)		26 (2.3)	2 (1.6)	
**Work situation**				< 0.001			< 0.001
Working fulltime, n (%)	44 (33.3)	442 (51.8)	150 (36.1)		548 (48.1)	44 (34.1)	
Working part-time, n (%)	11 (8.3)	125 (14.7)	38 (9.2)		156 (13.7)	7 (5.4)	
Retired, n (%)	55 (41.7)	166 (19.5)	177 (42.7)		281 (24.7)	62 (48.1)	
Other, n (%)	22 (16.7)	120 (14.1)	50 (12.1)		154 (13.5)	16 (12.4)	
**Body mass index**				< 0.001			< 0.001
< 25, n (%)	30 (22.7)	455 (53.2)	61 (14.6)		501 (43.8)	15 (11.6)	
25–30, n (%)	53 (40.2)	321 (37.5)	190 (45.6)		465 (40.7)	46 (35.7)	
> 30, n (%)	49 (37.1)	79 (9.2)	166 (39.8)		177 (15.5)	68 (52.7)	
**Waist circumference**				< 0.001			< 0.001
W < 80 M < 94, n (%)	19 (14.4)	412 (48.2)	10 (2.4)		422 (37.0)	0 (0.0)	
W80-88 M 94–102, n (%)	22 (16.7)	247 (28.9)	93 (22.3)		317 (27.8)	23 (17.8)	
W > 88 M > 102, n (%)	91 (68.9)	195 (22.8)	314 (75.3)		403 (35.3)	106 (82.2)	
**Physical exercise > 30 min/d**				< 0.001			< 0.001
Yes, n (%)	103 (79.8)	785 (93.0)	332 (80.6)		1021 (90.4)	96 (76.2)	
**Vegetables/fruits/berries**				0.683			0.812
Every day, n (%)	112 (84.9)	685 (80.8)	332 (79.8)		913 (80.4)	104 (81.3)	
**Have used anti-hypertensive drugs**				< 0.001			< 0.001
Yes, n (%)	83 (62.9)	726 (85.9)	182 (43.9)		264 (23.3)	88 (68.8)	
**Ever high blood glucose**				< 0.001			< 0.001
Yes, n (%)	118 (90.1)	16 (1.9)	71 (17.1)		38 (3.4)	49 (38.0)	
**Family history of diabetes**				< 0.001			< 0.001
Biological parents/siblings/children, n (%)	66 (50.4)	71 (8.4)	175 (42.1)		161 (14.2)	85 (65.9)	
Grandparents/ant/uncle/cousin, n (%)	23 (17.6)	148 (17.5)	97 (23.3)		222 (19.6)	23 (17.8)	
No, n (%)	42 (32.1)	629 (74.2)	144 (34.6)		752 (66.3)	21 (16.3)	
**FINDRISC score, mean (SD)**	17.1 (4.3)	5.8 (3.2)	13.7 (2.5)	< 0.001	7.6 (4.0)	17.7 (2.5)	< 0.001

T2D indicates self-reported type 2 diabetes; SD, standard deviation

^a^The Finnish Diabetes Risk Score (FINDRISC)

71 (5.1%) had missing on self-reported T2D, 14 (1.0%) on marital status, 4 (0.3%) on work situation. 1 (0.07%) on waist circumference, 19 (1.4%) on physical activity, 8 (0.6%) on vegetables/fruit/berries, 12 (0.9%) on have used anti-hypertensive drugs, 11 (0.8%) on ever high blood glucose, and 9 (0.6%) on family diabetes

^†^*P*-value: Comparison of FINDRISC categories (< 11 vs ≥ 11 and < 15 vs.** ≥ **15). Chi-square test for categorical data, t-test for continuous data

### Prevalence of persons with increased risk of developing T2D in general practice

We calculated the prevalence of individuals at risk of T2D by assessing FINDRISC scores (Table 2 and Supplemental Table 1). In Table 2, FINDRISC was categorized in two categories (< 11 vs ≥ 11, or < 15 vs ≥ 15). A total of 32.8% reported FINDRISC score ≥ 11. Corresponding number using a cut-off of 15 was 10.0% (FINDRISC ≥ 15). Overall, the prevalence of individuals at risk of T2D was comparable between men and women, but with more or less increasing differences with increasing age.

**Table 2:** Diabetes risk^a^ prevalence in 1272 Norwegians in general practice without a known diagnosis of diabetes

	**N (%)**	**FINDRISC**^a^** prevalence, % (95% CI)**	
		** ≥ 11**	** ≥ 15**
**All (95% CI)**	1272 (100.0)	32.8 (30.3–35.4)	10.0 (8.6–11.9)
**By groups**			
Sex			
Men	579 (45.5)	32.3 (28.6–36.2)	9.7 (7.5–12.4)
Women	693 (54.5)	33.2 (29.8–36.8)	10.5 (8.5–13.1)
Age categories			
All			
18–39	321 (25.2)	7.8 (5.3–11.3)	0.9 (0.3–2.9)
40–59	445 (35.0)	32.1 (28.0–36.6)	8.3 (6.1–11.3)
60–75	428 (33.7)	47.4 (42.7–52.2)	16.8 (13.6–20.7)
76–80	78 (6.1)	59.0 (47.8–69.3)	21.8 (14.0–32.3)
Men			
18–39	105 (31.2)	5.7 (2.6–12.2)	9.5 (1.3–6.5)
40–59	195 (33.7)	27.7 (21.9–34.4)	5.1 (2.8–9.3)
60–75	236 (40.8)	44.9 (38.7–51.3)	16.1 (11.9–21.4)
76–80	43 (7.4)	48.8 (34.4–63.5)	16.3 (8.0–30.4)
Women			
18–39	216 (31.2)	8.8 (5.7–13.4)	0.9 (0.2–3.6)
40–59	250 (27.7)	35.6 (29.9–41.7)	10.8 (7.5–15.3)
60–75	192 (27.7)	50.5 (43.5–57.5)	17.7 (12.9–23.8)
76–80	35 (5.1)	71.4 (54.5–83.9)	28.6 (16.1–45.5)

CI indicates confidence interval

^a^The Finnish Diabetes Risk Score (FINDRISC)

Of the 1272 persons without a known diagnosis of diabetes, 177 persons had FINDRISC score < 15 even though they reported two of the three following risk factors: Age > 50 years, BMI > 30 and/or family history of diabetes (parents/siblings/children). Eleven persons had all three risk factors, but still had a FINDRISC score < 15 (numbers not reported in tables).

In Supplemental Table 1 FINDRISC was categorized in 5 categories. One out of three individuals had FINDRISC score < 7, and 2 of 3 individuals had FINDRISC score < 11. Of the individuals with FINRDRISC score ≥ 11, only 10% scored ≥ 15 and less than 1% scored ≥ 20, the last score indicating a very high risk of diabetes the next 10 years.

**Fig. 1:**
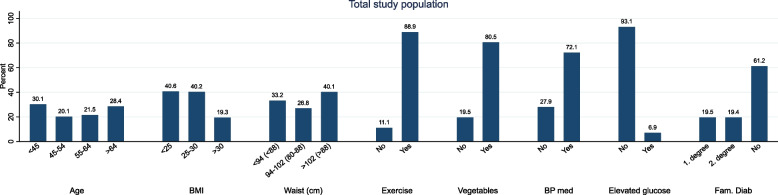
Percentages of the items of the Finnish Diabetes Risk Score (FINDRISC) in non-diabetic patients in general practice. Fam. Diab indicates family history of diabetes; BMI, body mass index; BP med., use of antihypertensive drugs. Cutoff-values for waist circumference in brackets are for women and outside brackets for men

In Fig. 1 we present the frequency distributions of the different items of the FINDRISC instrument in the non-diabetic population of patients. Approximately 60% had BMI ≥ 25, of which 19.3% had BMI > 30. Almost 90% reported exercising 30 min or more every day and 80% reporting eating vegetables every day. In Table 3 the results are stratified by sex. The frequency distribution of the items was quite similar between men and women; however, more women reported eating vegetables and fruits. Further, more women report having used anti-hypertensive drugs and to have a family history of diabetes.

**Table 3:** Frequency of the items of FINDRISC^a^ in the non-diabetic population in general practice

**FINDRISC**^a^** item**	**Men (*n* = 579)**	**Women (*n* = 693)**
Age		
< 45	131 (22.6)	252 (36.4)
45–54	105 (18.1)	150 (21.7)
55–64	147 (25.4)	126 (18.2)
> 64	196 (33.9)	165 (23.8)
Body mass index		
< 25	203 (35.1)	313 (45.2)
25–30	267 (46.1)	244 (35.2)
> 30	109 (18.8)	136 (19.6)
Waist circumference		
Men < 94; women < 80	184 (31.8)	238 (34.3)
Men 94–102; women 80–88	174 (30.0)	166 (24.0)
Men > 102; women > 88	220 (38.1)	289 (41.7)
Physical exercise > 30 min/day, Yes	511 (89.2)	606 (88.7)
Vegetables/fruits/berries, Yes	417 (72.7)	600 (87.0)
Have used anti-hypertensive drugs, Yes	380 (66.3)	528 (76.9)
Ever high blood glucose, Yes	28 (4.9)	59 (8.6)
Family history of diabetes		
Yes, first degree	91 (15.9)	155 (22.4)
Yes, second degree	94 (16.4)	151 (21.9)
No	388 (67.7)	385 (55.7)

^a^The Finnish Diabetes Risk Score (FINDRISC)

### Prevalence of self-reported T2D in general practice

We identified 132 participants with known diabetes. The prevalence of diabetes in the total study population were 9.9% (95% CI 8.4–11.6); 10.6% (95% CI 8.3–13.2) in men and 9.3% (95% CI 7.4–11.7) in women. The prevalence of diabetes increased with higher age (Supplemental Table 1).

## Discussion

In a population of 1272 individuals without a known diagnosis of diabetes, the prevalence of elevated FINDRISC score ≥ 11 and ≥ 15 was 32.8% and 10.0%, respectively. In general practice, FINDRISC, a simple, non-expensive and valid questionnaire, may be valuable as an initial screening method to identify those in need for preventive measures for diabetes. To identify manifest disease, the initial screening may be followed by a more accurate diagnostic tool assessment, such as measurement of glycated haemoglobin in blood (HbA1c).

Prevalence estimates of elevated FINDRISC like ours have been reported from comparable cross-sectional and cohort studies [20, 22, 24–26]. In our study 10.0% had FINDRISC score ≥ 15, reflecting a need for more healthy behaviors and life-style modifications. This is similar to the reports from the population-based HUNT study (n = 47,694) that observed 11% with FINDRISC ≥ 15 [25]. In the European Feel4Diabetes study with 2116 parents of primary-school children [27] the corresponding number was 12.8%. A study from Poland with 1090 participants [28] reported that 18.4% had a score ≥ 15, a higher number than expected that may be explained by overrepresentation of older and more obese participants. To our knowledge, few studies have assessed prevalence of elevated FINDRISC in a population that attended routine consultations in general practice, but a cross-sectional study (n = 11,444) of people 35 years or older at primary care settings in Europe found that 41.5% had elevated FINDRISC score ≥ 14 [29]. The corresponding number in our study was much lower; 14.5%. As FINDRISC is highly correlated with age this may partly explain the large difference in prevalence between this study and our study. Also, the participants in our study had a more favorable health profile with lower BMI, higher consumption of vegetables and more exercise. However, our participants also reported more family history of diabetes, use of anti-hypertensive drugs and previous measurement of high blood glucose. When recruiting from general practices, it is expected that the prevalence of people with co-morbidities is higher than in the general population. We did not have data on the participants’ diversity of diagnoses or reasons for seeing their general practitioner.

### The relevance of FINDRISC for risk assessment in general practice

Diabetes is a large global health problem, and a major challenge is to identify asymptomatic individuals and individuals at risk of diabetes. The rapid increase in the number of people with diabetes worldwide highlight the need for studies examining the usability of tools for risk-assessment. The advantage of using FINDRISC to detect people with elevated risk of developing diabetes lies in its simple and self-report format [30], in addition to being cost-effective. The last factor being especially important in developing countries. The long-lasting asymptomatic diabetes stage represents a challenge as diabetes-related complications may develop during this time. Among people at risk of developing diabetes a state of elevated glucose levels may persist for several years without reaching the diagnostic blood glucose levels of T2D. However, questions remain about the sensitivity and specificity of cutoff points [5, 18, 22, 27, 31]. Previous studies have shown that optimal cut-off points for T2D interventions vary widely [18, 31, 32]. Individuals in Norway with FINDRISC score ≥ 15 have been followed up with laboratory assessments and offered intensive low-threshold multifactorial lifestyle intervention if they had HbA1c or glucose levels indicating intermediate hyperglycemia [33]. Similar screening programs are also used in other European countries such as Denmark and Germany [34, 35]. It does, however, seem that routines for screening and follow-up of people at high risk of diabetes vary, which means that treatment to promote better risk-factor management for people at high risk of diabetes also differ. It may be clinical experience rather than guidelines that prompts screening [36]. In Norway, concerns have been raised about the sensitivity and specificity of the FINDRISC ≥ 15 threshold for the development of T2D. Many people who will develop diabetes over a 10-year period will not be captured using a FINDRISC cut-off of 15. But analysis show that lowering the definition of elevated FINDRISC score to ≥ 11 would identify 73% of the Norwegian population who subsequently will develop diabetes within the next 10 years [18]. This would substantially impact the resources in general practice [18]. Of the 1404 men and women in the current study, 324 had a FINDRISC score > 11, and, 129 (10%) had FINDRISC score ≥ 15. A European study found cut-off ≥ 14 to be most suitable for identifying undiagnosed T2D, while ≥ 12 proved to be optimal for detection of dysglycemia [27]. The ideal cut-off seems to differ according to several aspects such as country and ethnicity, and it may be useful to customize FINDRISC to different populations.

HbA1c ≥ 48 mmol/mol (≥ 6.5%) is the accepted method used to diagnose diabetes, and in asymptomatic individuals it is recommended to repeat the test [37]. However, there will always be a trade-off between simplicity and accuracy for different screening methods. To reach those that may benefit from counselling interventions to promote healthier decision making at an earlier stage might lead to more efficient lifestyle follow-up programs. In line with guidelines and previous studies, we suggest continuing using a two-step approach to identify those at high risk of diabetes and those with manifest disease 1) a simple first-level screening to identify those in need for preventive health guiding and support, followed by 2) a more accurate diagnostic tool assessment (HbA1c) to identify undiagnosed diabetes [27, 38]. FINDRISC is such a simple and non-invasive diabetes risk score which can be well understood by lay people and clinical personnel without any laboratory test. The instrument might assist healthcare professionals to identify those with modifiable risk factors among people with complex and demanding health issues that is frequent in general practice. Nevertheless, given that time is a limited resource in general practice, simplifying the FINDRISC score may improve its efficiency. Simplified tools applied in other countries such as Germany [39] (including age, BMI, history of high blood glucose) and Spain [31] (including BMI, use of antihypertensive medication, history of high blood glucose) performed equally and better than the original FINDRISC in detecting undiagnosed T2D. More studies testing the validity, as well as sensitivity and specificity of shorter form versions of risk assessment tools are warranted. FINDRISC can contribute to early detection of individuals at risk of developing diabetes and those with undiagnosed diabetes, and also, help reduce the burden of diabetes complications. We do, however, recognize the limitation of the FINDRISC score alone to detect glucose alterations and unknown diabetes.

Knowledge on the screening practices for diabetes done by GPs` are scarce. It has been found that < 10% of people at high risk of diabetes are aware of it [40], and several studies show that physicians do not aggressively screen and treat high risk individuals to stop progression to diabetes and to prevent complications [41, 42]. This may be attributed to the overburden of preventive tasks that are reported by GPs` and nurses in general practice [43–45]. FINDRISC with its simple and non-invasive format is still relevant in GP consultations, as it may be used independent of a regular GP appointment and also outside the GP`s office, e.g., through websites, at home or at pharmacies. The shift to paying more attention to inadequate health behaviors and health promotion strategies in primary health care is a question of priorities that must be handled by leaders and politicians.

### Strengths and limitations

The strength of this study includes a high participation rate, which enabled precise estimates of prevalence of individuals at high risk of T2D in general practice. We have also used an established and validated risk assessment tool, FINDRISC, when estimating diabetes risk in individuals without a known diabetes diagnosis. Also, the inclusion of all persons 20–80 years of age in the waiting room area indicates that our results are representative for the general population attending a GP appointment. Some limitations need to be addressed. First, diabetes and several other factors were self-reported, which means that the estimates may be influenced by clinical, psychological, and behavioural factors. It is, i.e., more likely that women have a history of measured high blood glucose due to standardized follow-up program during pregnancy, which further will impact the overall FINDRISC score. Of those reporting that they had diabetes the diagnosis was confirmed by checking the medical records. Use of anti-hypertensive medication was reported by over 70% of the study population, but not verified by medical records. This might indicate that this “waiting-room” population have more chronic disease. We lacked information on diabetes type, duration and glycaemic control (HbA1c). Lack of information on HbA1c also precludes the opportunity to examine the validity of FINDRISC. Recall bias is a potential risk in studies like this, as people may report unprecise lifestyle estimates and therefore generate concern over the true accuracy of the respondents self-reporting of health events. Lastly, the cross-sectional design of this study precludes causality.

## Conclusion

Routine screening with the FINDRISC questionnaire in general practice showed that a significant proportion of patients attending a consultation with a GP had modifiable obesity-related problems. Detection of individuals with disadvantageous BMI, waist circumference, and level of physical activity may be of high public health relevance to intensify early implementation of preventive health care programs aimed to reduce the risk of diabetes and its complications through lifestyle modification. The higher proportion of patients with a family history of diabetes among those with a FINDRISC score above 15 further illuminates the need for identification and support to facilitate a healthier lifestyle among those at risk.

## Supplementary Information

**Additional file 1: Supplemental Table 1.** Diabetes risk* prevalence in 1272 Norwegians in general practice without a known diagnosis of diabetes.

## Data Availability

The dataset used for the current study is available from the corresponding author on reasonable request.

## Abbreviations

T2D: Type 2 diabetes
FINDRISC: The Finnish Diabetes Risk Score
BMI: Body mass index
SD: Standard deviation
CI: Confidence interval
GP: General practitioner
HbA1c: Hemoglobin A1c
REK: Regional Committee for Medical and Health Research Ethics
NSD: The Norwegian Centre for Research Data
