# Supplementary material for: Prevalence of increased risk of type 2 diabetes in general practice: a cross-sectional study in Norway
Source: BMC Prim Care. 2023 Jul 20;24:151. doi: 10.1186/s12875-023-02100-x (PMC10357693; doi:10.1186/s12875-023-02100-x)
Supplement: Supplementary file 1 — Additional file 1: Supplemental Table 1. Diabetes risk* prevalence in 1272 Norwegians in general practice without a known diagnosis of diabetes. [file 12875_2023_2100_MOESM1_ESM.docx]

Supplemental material

**Supplemental Table 1:** Diabetes risk* prevalence in 1272 Norwegians in general practice without a known diagnosis of diabetes

|  | **N (%)** | **T2D prevalence** | **N (%)** | **FINDRISC* Prevalence (95% CI)** | | | | |
| --- | --- | --- | --- | --- | --- | --- | --- | --- |
|  |  |  |  | **<7** | **7-10** | **11-14** | **15-19** | **≥20** |
| **All** | 1404 (100.0) | 9.9 (8.4-11.6) | 1272 (100.0) | 33.5 (30.9-36.1) | 33.7 (31.2-36.4) | 22.6 (20.4-25.0) | 9.2 (7.7-10.9) | 0.9 (0.5-1.7) |
| **By groups** |  |  |  |  |  |  |  |  |
| Sex |  |  |  |  |  |  |  |  |
| Men | 644 (45.9) | 10.6 (8.3-13.2) | 579 (45.5) | 29.9 (26.3-33.7) | 37.8 (34.0-41.9) | 22.6 (9.4-26.2) | 9.3 (7.2-12.0) | 0.4 (0.1-2.4) |
| Women | 760 (54.1) | 9.3 (7.4-11.7) | 693 (54.5) | 36.5 (33.0-40.2) | 30.3 (27.0-33.8) | 22.7 (19.7-25.9) | 9.1 (7.2-11.5) | 1.4 (0.8-2.7) |
| Age |  |  |  |  |  |  |  |  |
| 18-39 | 327 (23.3) | 1.9 (0.8-4.2) | 321 (25.2) | 70.7 (65.5-75.4) | 21.5 (17.3-26.3) | 6.9 (4.6-10.2) | 0.9 (0.3-2.6) | - |
| 40-59 | 490 (34.9) | 9.7 (7.4-13.0) | 445 (35.0) | 30.3 (26.2-34.8) | 37.5 (33-1-42.1) | 23.8 (20.1-28.0) | 6.7 (4.8-9.5) | 1.6 (0.8-3.3) |
| 60-75 | 494 (35.2) | 13.8 (11.0-17.2) | 428 (33.7) | 12.1 (9.4-15.6) | 40.4 (35.9-45.1) | 30.6 (26.4-35.1) | 16.1 (12.9-19.9) | 0.7 (0.2-2.2) |
| 76-80 | 93 (6.6) | 16.5 (10.2-25.6) | 78 (6.1) | 15.4 (8.9-24.2) | 25.6 (17.2-36.4) | 37.2 (27.2-48.4) | 19.2 (11.9-29.5) | 2.5 (0.6-9.7) |

T2D indicates type 2 diabetes mellitus

*The Finnish Diabetes Risk Score (FINDRISC)
